# Supplementary material for: Domains and Categories of Needs in Long-Term Follow-Up of Adult Cancer Survivors: A Scoping Review of Systematic Reviews
Source: Healthcare (Basel). 2024 May 22;12(11):1058. doi: 10.3390/healthcare12111058 (PMC11172118; doi:10.3390/healthcare12111058)
Supplement: Supplementary file 1 [file healthcare-12-01058-s001.zip › healthcare-2957418-supplementary.pdf]

Review

Domains and Categories of Needs in Long-Term Follow-Up of Adult Cancer Survivors: A Scoping Review of Systematic Reviews

Nicolas Sperisen [1,2,*], Dimitri Kohler [2], Nicole Steck [2], Pierre-Yves Dietrich [3,4] and Elisabetta Rapiti [5]

1 Institute of Global Health, Faculty of Medicine, University of Geneva, 1205 Geneva, Switzerland
2 Swiss Cancer League, 3001 Bern, Switzerland; dimitri.kohler@gmail.com (D.K.); nicole.steck@krebsliga.ch (N.S.)
3 Clinique des Grangettes, Hirslanden, 1224 Geneva, Switzerland; pierre-yves.dietrich@hirslanden.ch
4 Faculty of Medicine, University of Geneva, 1205 Geneva, Switzerland
5 Geneva Cancer Registry, Institute of Global Health, Faculty of Medicine, University of Geneva, 1205 Geneva, Switzerland; elisabetta.rapiti@unige.ch
* Correspondence: nicolas.sperisen@krebsliga.ch; Tel.: +41-313-899-290

TABLE S1. Results According to Research Questions.

Authors (year)	Identified needs (domains)	Socio-demographic factors associated with needs	Greater needs in the transition phase
Hoekstra et al. (2014) [24]	▪ Medical<br>▪ Psychosocial<br>▪ Information<br>▪ Proactive contact<br>▪ Other	-	▪ Proactive approach of the general practitioner
Hyun et al. (2016) [21]	Information on:<br>▪ Thyroid cancer<br>▪ Thyroid cancer treatment<br>▪ Diagnostic tests<br>▪ Aftercare<br>▪ Psychosocial issues<br>▪ Coordination of care<br>▪ Complementary and alternative medicine	-	-
Kotronoulas et al. (2017) [19]	▪ Physical / cognitive<br>▪ Psychosocial / emotional<br>▪ Family-related<br>▪ Social / societal<br>▪ Interpersonal / intimacy<br>▪ Practical / daily living<br>▪ Information / education<br>▪ Health system / patient-clinician communication needs	▪ Gender<br>▪ Age<br>▪ Education level<br>▪ Employment status<br>▪ Family support	▪ Better coordination among healthcare professionals<br>▪ Psychological support for feeling of abandonment
Lehmann et al. (2021) [14]	Sexual health-related care / Sex-related:<br>▪ Information<br>▪ Practical / emotional support<br>▪ Communication	▪ Age<br>▪ Gender<br>▪ Relationship status	-
Lim et al. (2021) [25]	▪ Physical symptoms<br>▪ Functional limitations	-	▪ Long-term support

	▪ Psychosocial impacts<br>▪ Financial impacts<br>▪ Interaction with healthcare system<br>▪ Coping<br>▪ Positive outcome		▪ Support for feeling of abandonment by the healthcare team
Lisy et al. (2019) [26]	▪ Psychosocial<br>▪ Supportive care<br>▪ Physical	▪ Age<br>▪ Education level<br>▪ Employment status<br>▪ Social support	▪ Support for anxiety about leaving the hospital system
Maguire et al. (2015) [20]	▪ Physical<br>▪ Psychological / emotional<br>▪ Social<br>▪ Interpersonal / intimacy concerns<br>▪ Health system / information<br>▪ Patient-clinician communication<br>▪ Spiritual / existential	-	▪ Support about intimate relationships<br>▪ More information regarding prognosis<br>▪ Better communication with the clinical team
Miroševič et al. (2019) [22]	▪ Psychological<br>▪ Physical and daily living,<br>▪ Relationship<br>▪ Patient care<br>▪ Information	▪ Age<br>▪ Employment status[a]<br>▪ Education level[a]<br>▪ Social support[a]<br><br>[a] weak evidence	▪ Support for fear of cancer recurrence<br>▪ Better information<br>▪ Reassurance about being treated
Pape et al. (2021) [27]	Before surgery (stoma reversal):<br>▪ Information before surgery<br>▪ Sources of information<br><br>After surgery:<br>▪ Management and coping<br>▪ Support from peers and the environment	-	-

	▪ Support of the healthcare professionals		
Van der Kruk et al. (2021) [28]	▪ Financial and travel issues<br>▪ Accessibility to care<br>▪ Psychological<br>▪ Information	▪ Location (urban vs rural)<br>▪ Education level<br>▪ Age<br>▪ Income	-
